# The Role of aDNA in Understanding the Coevolutionary Patterns of Human Sexually Transmitted Infections

**DOI:** 10.3390/genes9070317

**Published:** 2018-06-25

**Authors:** Ville N. Pimenoff, Charlotte J. Houldcroft, Riaan F. Rifkin, Simon Underdown

**Affiliations:** 1 Department of Cancer Epidemiology and Prevention, Bellvitge Institute of Biomedical Research, Catalan Institute of Oncology, 08908 Barcelona, Spain; 2 Department of Archaeology, University of Helsinki, 00014 Helsinki, Finland; 3 Department of Epidemiology, University of Tampere, FI-33014 Tampere, Finland; 4 Department of Medicine, Addenbrookes Hospital, University of Cambridge, Cambridge CB2 0QQ, UK; ch504@cam.ac.uk; 5 Department of Archaeology, University of Cambridge, Cambridge CB2 3QG, UK; 6 Parasites and Microbes, Wellcome Sanger Institute, Hinxton CB10 1SA, UK; 7 Centre for Microbial Ecology and Genomics, Department of Biochemistry, Genetics and Microbiology, University of Pretoria, Hatfield 0028, South Africa; riaanrifkin@gmail.com; 8 Human Origins and Palaeo-Environments Research Group, Department of Anthropology and Geography, Oxford Brookes University, Oxford OX3 0BP, UK; sunderdown@brookes.ac.uk

**Keywords:** evolutionary medicine, sexually transmitted infections, papillomaviruses, herpesviruses, ectoparasites, virus-host coevolution, divergence, host-switch, Hominin evolution

## Abstract

Analysis of pathogen genome data sequenced from clinical and historical samples has made it possible to perform phylogenetic analyses of sexually transmitted infections on a global scale, and to estimate the diversity, distribution, and coevolutionary host relationships of these pathogens, providing insights into pathogen emergence and disease prevention. Deep-sequenced pathogen genomes from clinical studies and ancient samples yield estimates of within-host and between-host evolutionary rates and provide data on changes in pathogen genomic stability and evolutionary responses. Here we examine three groups of pathogens transmitted mainly through sexual contact between modern humans to provide insight into ancient human behavior and history with their pathogens. Exploring ancient pathogen genomic divergence and the ancient viral-host parallel evolutionary histories will help us to reconstruct the origin of present-day geographical distribution and diversity of clinical pathogen infections, and will hopefully allow us to foresee possible environmentally induced pathogen evolutionary responses. Lastly, we emphasize that ancient pathogen DNA research should be combined with modern clinical pathogen data, and be equitable and provide advantages for all researchers worldwide, e.g., through shared data.

## 1. Introduction

Every human carries infectious agents during their lifetime, including sexually transmitted microbes. Accumulating viral pathogen genome data sequenced from clinical samples has made it possible to infer phylogenetic analysis of sexually transmitted infections on a global scale and to estimate the diversity and distribution of these pathogens, and their common evolutionary histories with their host [1,2]. In parallel, high throughput sequencing technologies, along with sequence capture enrichment methods, have made the analysis of ancient genetic material possible through the retrieval of endogenous human and pathogen DNA from ancient samples [3,4,5].

Viruses and other pathogens commonly spread through anogenital or oral contact have a long-standing association with humans. Most of these sexually transmitted pathogens, either as persistent or acute infections [6], are carried through life. They mostly mutate slowly and spread mainly from person to person through sexual contact [7,8]. Although mammalian reproductive behaviors can be quite complex, it is commonly assumed that human sexual behaviors are ubiquitous [9,10]. Therefore, it is very likely that archaic hominins carried several sexually transmitted infections (STIs), and some of them were inherited from their ancestors [1,11,12,13]. Nevertheless, a note of caution is needed as nonsexual transmission of modern STIs may have played a role in ancient pathogen transmission.

The analysis of pathogen genome diversity provides significant insights into pathogen emergence and disease prevention [14]. Using deep-sequenced pathogen genomes retrieved from clinical studies [2], it is possible to estimate the within-host and between-host evolutionary rates, and to understand the changes in genomic stability and evolutionary responses for a particular pathogen [15]. Furthermore, applying substitution rates in ‘measurably evolving’ pathogen genome data [16] enables the generation of the minimum divergence time estimate of the pathogen [17]. Subsequently, the population’s genetic structure provides data on the population structure of the virus [18], and the host, if codivergence is assumed [14]. Thus, particular pathogens hold complementary stories about human evolutionary history. The genomes of slowly evolving STIs may even help us to understand when and where hominins had sex, as not all sexual interactions led to successful interbreeding.

**Figure 1:**
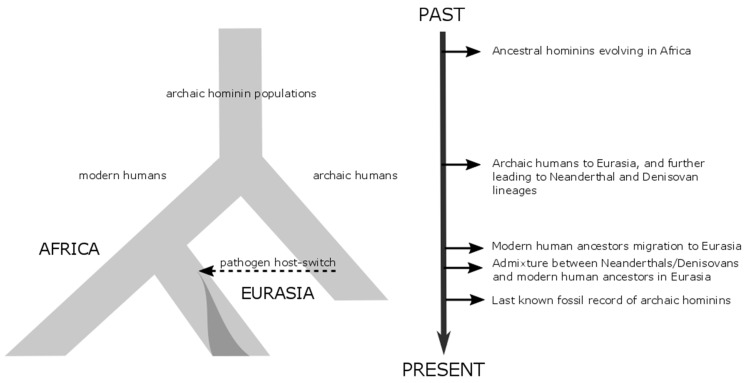
Hominin host-switch model (modified from [1]). Schematic diagram of the pathogen evolution and distribution out-of-Africa with the corresponding ancestral hominin group. Since the migration of modern human ancestors to Eurasia 120,000–60,000 years ago, sexual transmission from Neanderthals, Denisovans, or other hominin groups (i.e., introgression and pathogen host-switch) likely introduced certain human papillomavirus, herpesvirus, and ectoparasitic pathogens to the arriving group of modern human ancestors in Eurasia.

Recently retrieved hominin fossils [19,20,21] and ancient hominin genomes [22,23,24] have transformed our view of human evolution over the last 500,000 years by revealing that our modern human ancestors formed part of a network of archaic hominin populations linked by significant, although fluctuating, gene flow (Figure 1) [25]. The evolutionary relationship is still unclear between our closest archaic and modern human relatives (i.e., Denisovans, Neanderthals, and archaic Africans) and the time frame of their likely introgression within the African archaic hominin metapopulation, including modern human ancestors [21,25,26,27]. In this context, we still have enormous gaps in our understanding not only of archaic African human dispersals, but also the origins and impact of infectious diseases on human evolution in Africa before modern human ‘Out-of-Africa’ migrations [21,28].

The emergence of hominins in Africa was influenced by the diversity and wide distribution of human pathogens [29,30]. From the total of 1415 human pathogens known today, 61% are zoonotic and 175 cause infectious diseases, several of which originate from Africa [29,30,31,32,33,34,35]. In particular, these ancient African pathogens share the longest common evolutionary history with hominins prior to their out-of-Africa migrations, and thus presumably constrained the hominin populations already in Africa [29,30,31,33,34,35,36]. Such patterns of pathogen-stress constraining the host population has been observed in hunter-gatherer populations in sub-Saharan Africa [34,35,37,38,39,40].

**Figure 2:**
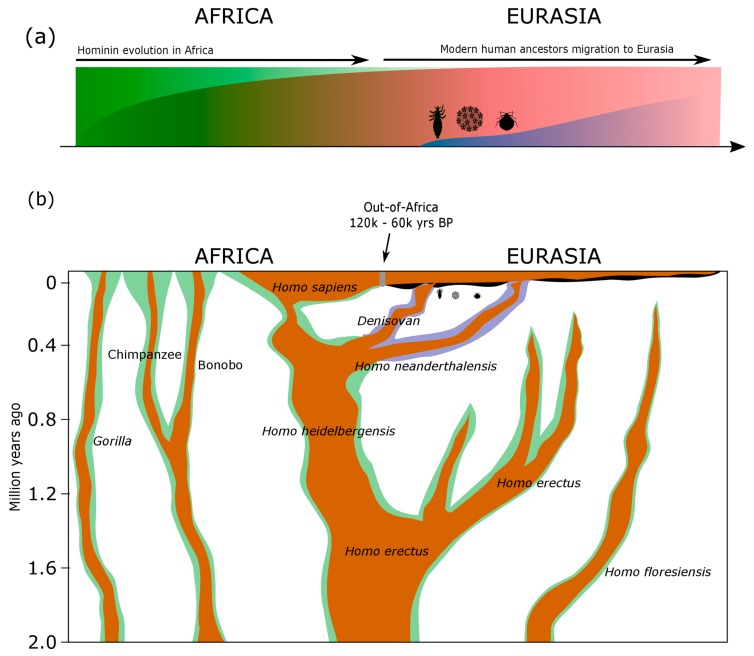
Human pathogen origin and transmission scenario between modern human ancestors, other hominin groups, and the African great apes. (**a**) Schematic diagram as a function of time (*x*-axis) of the human pathogen presence originating from Africa (e.g., human papillomavirus (HPV), herpes simplex virus (HSV), and human immunodeficiency virus (HIV) [29,30,33] depicted in green) and evolving among modern humans migrating out-of-Africa (red color), with additional transmission between modern humans and other hominins in Eurasia (e.g., HPV [1], HSV [42], lice [44], and bed bugs depicted as three icons and subsequent prevalence in blue color). Hence, we highlight that, following the out-of-Africa migration of modern humans, sexual transmission from Neanderthals or other hominin groups likely introduced certain HPV, HSV, and ectoparasitic pathogens to modern humans, many of which are still prevalent today. (**b**) The majority of modern human pathogens emerged in Africa (illustrated as green shades along the corresponding Hominin and great ape evolutionary tree [29,30,33]), where they were transmitted between ancestral hominin groups and the African great apes, and further codiverged and dispersed outside Africa with the corresponding ancestral hominin groups: evolution of Neanderthal and Denisovan populations exclusively in Eurasia with their corresponding pathogens (illustrated in blue color and with three icons ca. 800–400 kya [48,49,50,51]), also leading to host-switch events with modern human ancestors after modern human out-of-Africa migration and admixture, ca. 120–60 kya [48,49]. Pathogens associated with modern humans outside Africa are illustrated in blackshades along the corresponding modern human dispersals. Hominin evolution with wavy branch edges indicating presumed population fluctuations in time was modified from [52].

This new understanding of a more complex human evolutionary history challenges prevailing epidemiological hypotheses that human infectious diseases only started to severely impact humans after the emergence of agriculture and sedentary lifestyles [33,41]. Instead, it advocates an ancient pathogen origin and transmission scenario, including host-switch events typical of pathogen evolution, between ancestral hominin groups and the African great apes (Figure 2) [1,12,42,43,44,45,46,47].

Molecular analytical techniques are increasingly applied to the field of pathogen ancient DNA (aDNA), and the results contribute significantly to our understanding of prehistoric epidemiology [53,54,55]. For example, given the ambiguities involved in assigning *Mycobacterium tuberculosis* or *Brucella melitensis* as causative agents of macromorphological skeletal features, the biomolecular (DNA) analysis of archaeological human remains has gained increasing recognition [17,56].

Here we examine questions related to three groups of pathogens transmitted mainly through sexual contact between modern humans, which are likely to provide insight into ancient human behavior and history. We especially focus on the *Papillomaviridae*, *Herpesviridae,* and ectoparasitic pathogen families.

## 2. Human Papillomaviruses

Papillomaviruses (PVs) are a diverse family of double-stranded DNA viruses with maybe 500 million years of common evolutionary history with vertebrates [57,58]. More than 350 PVs have been identified from a range of host species, from fish to mammals, and over 200 of them are human papillomaviruses (HPVs) [58,59]. Based on host resources [60], the evolution of mammalian PVs started with environmentally-directed diversification and resulted in a few viral crown groups associated with the evolution of mammalian fur and skin glands [61,62]. Subsequently PV evolution was driven by the codivergence with the mammalian adaptive radiation, however, incomplete lineage sorting and host-switch events leading to lost or newly distributed lineages, respectively, also played a significant role in the evolution of PVs [47,57,63,64]. Assuming limited virus-host coevolution, the evolutionary rate for PVs has been estimated around 10^−8^ substitutions per site per year [57,65]. This fits our knowledge of PV lifestyle, as PVs do not encode for DNA polymerases and resort instead for replication to high-fidelity host nuclear polymerases [66]. However, these rate estimates should be interpreted with caution because a priori assuming host-pathogen codivergence as a leading drive of PVs evolution might lead to factitious estimates as demonstrated for human polyomaviruses [67].

HPVs infect basal epithelial cells at the cutaneous and mucosal sites, and a fraction of some 50 HPVs are found in the genital tract [68]. These HPVs are the most common sexually transmitted infections in humans, and practically all sexually active individuals become infected by a number of anogenital HPVs. Fortunately, most sexually transmitted HPV infections remain asymptomatic and are cleared by the host immune system [69]. Prevalence of these transient HPV infections is estimated at 12% in healthy women worldwide [70]. However, a persistent anogenital infection by a subset of at least twelve oncogenic HPVs is associated with the development of genital and anal cancers [68].

HPVs have coexisted with humans since the origin of our species, and with variable clinical manifestations, the globally circulating HPV population has been largely shaped by host population-level processes [14,71]. The continuous interplay with the differential host immune response among humans has likely further constrained the population structure of the HPVs [14]. It was previously assumed that HPVs coevolved exclusively among modern humans, but recent work using Bayesian phylogenetic inference suggests that HPVs evolved separately within each archaic hominin population, and subsequent interbreeding between Neanderthals and ancestral modern humans led to pervasive host-switching of HPVs (Figure 1) [1]. A comprehensive HPV16 phylogeography reveals a clear geographic structure and a deep split between the two main HPV16 lineages (HPV16A and HPV16BCD) [1]. Testing the two opposing models, the *recent-out-of-Africa* and *Hominin-host-switch* models, for HPV16 coevolution showed that codivergence exclusively with modern humans explains at most 30% of the observed global HPV16 diversity distribution. Instead, the most likely explanation of the deep HPV16 divergence and phylogeography was the *Hominin-host-switch* model (Figure 1), which required the basal HPV16A lineage transmission from archaic to modern humans after their migration to Eurasia [1].

These findings could explain an unusual aspect of HPV genetic diversity: why is the HPV16A variant virtually absent in sub-Saharan Africa when it is the most common outside Africa? Collectively, all ancestral hominin populations in Africa were likely infected by similar archaic HPVs. Hence, when Neanderthal and Denisovan populations evolved in Eurasia some 400,000–800,000 years ago, they initially carried a similar archaic collection of HPV16 diversity as their archaic contemporaries, but which subsequently evolved into a distinct collection of Neanderthal/Denisovan HPV16 variation, and ultimately led to the HPV16A lineage (see also Figure 2).

When the ancestors of modern humans migrated out of Africa some 60,000–120,000 years ago, they carried their HPV16 diversity, namely the HPV16BCD lineages, which initially evolved among modern human ancestors in Africa. Repeated interbreeding between Neanderthals and modern human ancestors in Eurasia transmitted the HPV16A lineage from archaic populations to modern human ancestors [1]. Furthermore, a subsequent study of oncogenic HPV58 diversity concluded the *Hominin-host-switch* model was the most likely explanation for the deep HPV58 divergence [46]. An additional evaluation, however, for the HPV58 phylogenetic inference may be needed as neither the effect of natural selection nor divergence model were comprehensively tested [72,73].

To independently explore ancient HPV diversity patterns, including divergence in evolutionary timescales, requires the retrieval of endogenous HPV DNA from ancient tissue samples. Pimenoff and colleagues [1] examined available archaic hominin preassembly sequence data, but no endogenous HPV DNA was retrieved. HPVs exclusively infect basal epithelia that are not likely to be preserved in ancient bone samples. Cutaneous epithelia-infecting HPVs are naturally present in all of us and typically found in most human metagenomic data [74]. It is theoretically possible, however, there seems to be no evidence of HPV DNA with a clear ancient DNA decay pattern thus far, from ancient or archaic human sample [75]. Likewise, a recent study claiming successful retrieval of 27,000 year old rodent PV DNA critically needs further confirmation using deep sequencing [76].

To sequence HPV DNA from paleopathological samples, visible HPV-induced warts offer an alternative. A recent study has reported the retrieval of short ~140bp DNA fragments of HPV18 from a 16th century mummified wart sample [77]. However, caution is needed as the HPV18 sequences retrieved were not published, and the result has not yet been confirmed. Future studies using aDNA enrichment technology targeted for HPVs and applied to deep sequencing of well-preserved ancient human tissue samples could enable an independent analysis of the deep coevolutionary patterns of sexually transmitted HPV infections in humans. Also, future HPV genome sequencing from nonvaccinated and vaccinated individuals worldwide with normal cytological findings will likely reveal a more accurate estimate of the phylodynamics of HPVs in modern humans.

## 3. Human Herpesviruses

Herpesviruses are ancient, double-stranded DNA viruses with large genomes which infect a range of metazoans, from corals to mammals [78,79]. Herpesviruses are associated with diverse disease manifestations, from asymptomatic primary infection, to blistering and cancers. They are predominantly spread through close physical contact and exchange of bodily fluids, including breast-feeding and sexual intercourse [80,81,82].

With the increasing availability of whole herpesvirus genomes from humans and other mammals [2,83,84,85], there is growing awareness that primate herpesviruses can illuminate hominin evolution, particularly interactions between hominin species who may not be the direct ancestors of living humans [43,83]. A study of chimpanzee simplex and human herpes simplex viruses (HSV) 1 and 2 found that chimpanzee simplex and HSV1 cospeciated with their respective hosts. HSV2, the aetiological agent of genital herpes in humans, originated as a cross-species transmission of chimpanzee simplex into the human lineage millions of years after the divergence of the last common ancestor of humans and chimpanzees [43]. Modelling suggests that HSV2 was transmitted from the ancestors of chimpanzees to *Homo erectus* by *Paranthropus boisei* [42]. These inferences were drawn from viruses collected from living primates, but our knowledge is incomplete and we do not currently have access to sequences of the α-herpesviruses which infect gorillas and bonobos. These may reveal whether cross-species transmission of α-herpesviruses is common amongst the great apes. Others have speculated that hominin-to-hominin transmission of a human α-herpesvirus, varicella-zoster virus, may have contributed to the extinction of the Neanderthals [86].

The HSV2 cross-species transmission event probably took place 2 mya, beyond the current event horizon for aDNA preservation in even the most favorable conditions. Furthermore, human α-herpesviruses are not oncogenic and would not preserve in tumor samples, although preserved oral soft tissue samples or blood within teeth from more recently deceased humans could be a source of α-herpesvirus aDNA.

There are a number of questions concerning the evolution of the first oncogenic human virus to be identified, Epstein-Barr virus (EBV) [87], and related oncogenic human γ-herpesvirus KSHV [88]. EBV is grouped into two types, 1 and 2 [89], with genotypic and phenotypic differences in latency associated genes (EBNAs) which are key for the virus’ persistence within the host following primary infection [87,90]. Differences within EBNA genes are also associated with differences in tropism between type 1 (B cells) and type 2 (T cells [91]) EBV. It is currently unknown when EBV types 1 and 2 diverged. Differences beyond the known EBNA-2 and EBNA-3A/B/C cluster are not linked to EBV type, pointing to a common origin for the rest of the EBV genome as a single species [89]. The divergence of type 1 and 2 EBNA gene clusters may be linked to human evolutionary history, given the lower prevalence and differing distribution of type 2 outside Africa [92]. Evidence for cross-species transmission and recombination in the KSHV genome [93] raises the possibility that EBV may also have an unusual evolutionary history. Modern clinical sequence data could address this by providing more sequences from modern and historical African samples [2] for evolutionary analysis [1,94]. There is scope for aDNA to answer these questions. As both human γ-herpesviruses are oncogenic, metastatic EBV or KSHV+ tumors would be macroscopically visible as skeletal pathology in the form of neoplastic lesions in the osteogenic mesenchyme and contain copies of the integrated viral DNA within the tumor cells. Mummified human remains are another potential source, as is dental pulp [95].

## 4. Human Ectoparasites

One of the most challenging aspects of understanding ancient human health is determining when diseases emerged and how they spread. Haematophagous ectoparasitic arthropods (e.g., lice and bedbugs) are among the oldest human parasites and, being dependent on their human hosts for mobility, represent excellent markers of ancient human migrations. As vectors of lethal pathogens, including epidemic typhus (*Rickettsia prowazekii*), trench fever (*Bartonella quintana*), and relapsing fever (*Borrelia recurrentis*), lice and bedbugs are of medical importance. Indeed, resembling ancient human dispersal patterns [96], molecular analyses of microbial pathogens [34,35] support a serial founder effect model *out-of-Africa*. Similarly, conspecific human parasites (e.g., human lice) have been used to trace the migration of humans from Africa and into the New World [97].

Humans are parasitised by two species of lice, pubic lice, *Pthirus pubis*, and head and body lice, *Pediculus humanus*. Human pubic or ‘crab’ lice share many morphological similarities and a most recent common ancestor with the gorilla louse, *Pthirus gorillae*. Based on mitochondrial DNA (mtDNA) sequence data, *P. gorillae* and *P. pubis* are estimated to have diverged some 3.3 mya [98]. As the *Pthirus* life cycle does not include a free-living phase, the *Pthirus* host species transfer implies spatio-temporal overlap and close physical contact between hominins and ancestral gorillas. At this time, the sub-Saharan African rainforest margins were inhabited by at least three candidate species of ancestral humans which could have facilitated the transfer of *P. pubis* to either *H. habilis* or *H. erectus*. While the appearance of *Australopithecus africanus* at ca. 3.0 mya might represent a good fit for the estimated divergence date of c. 3.3 mya, the presence of *Australopithecus afarensis* (from c. 3.9 to 2.8 mya) at the margins of the African rainforests must also be considered. Once *Pthirus* reached *H. erectus*, no further host-switches were required for *Pthirus* to continue its close association with hominins in the lineage leading to modern *H. sapiens*.

While human ectoparasites such as lice can survive for thousands of years in archaeological contexts (e.g., [97]), morphological identification is problematic as remains are often highly fragmented. The analyses of mitochondrial DNA of ancient head louse eggs from Israel suggests close affinities with lice of people originating in west Africa [99]. In the near future, genome data derived from ancient African contexts, in combination with probability-based network analysis [42], might provide significant insight into the origins, transmission, and persistence of these undesirable passengers.

## 5. Ancient Pathogen DNA Prospects

Currently circulating viral and ectoparasitic diversity may only represent the most successful or recent strains (strain replacement), which often capture a fraction of the genetic diversity of the particular pathogen’s evolutionary history and can cause factitious divergence time estimates [100]. Recently sampled microorganisms may lack sufficient temporal structure and lead to spurious evolutionary rate estimates [101]. Most importantly, molecular evolution rates are shown to be time-dependent and without sufficient independent calibration points, which pathogen aDNA can provide, within the time depths of the analysis the divergence time estimates can be systematically overestimated [3,17,102,103,104,105]. However for some rapidly evolving viruses such as hepatitis B [106], aDNA calibration points do not appear to improve the accuracy of estimates of the age of currently circulating viral diversity [107]. Recent studies of HBV aDNA have produced conflicting results as to whether molecular clock-based approaches to dating how long HBV has been a human pathogen are suitable [106,108,109]. They have also emphasized the power of direct dating of HBV from ancient human remains to demonstrate how long HBV has infected humans, its diversity and distribution in past populations, and the possibility of transmission from nonhuman primates [108,109]. Another issue in molecular dating of pathogen origins and divergence is cross-validation [110] to evaluate the differential fit of the alternative models [72,111,112]. These studies further raise the prospect that HBV may have been spread by nonsexual routes during the Pleistocene.

We consider that preserved anatomical specimens are an excellent source from which to extract ancient pathogen DNA, particularly viruses and intracellular pathogens. Both disease lesions [113] and cancer specimens [114] of increasing age have been successfully sequenced and have the scope to include pathogen DNA. Museum collections also represent vital repositories of ancient host and pathogen DNA [115,116,117]. If macroscopic samples of soft tissue or bone are not preserved, then sedimentary ancient DNA can yield ancient pathogen DNA sequences. By employing the techniques of modern environmental sampling [118] and skeletal aDNA analysis [23], ancient pathogen DNA can be successfully recovered from coprolites [119], museum specimens [120], and prehistoric sediments [121,122,123].

## 6. Ancient DNA from Anthropogenic Sediments?

Environmental DNA (eDNA) is genetic material obtained from aquatic or terrestrial sediments and marine or fresh water which do not contain identifiable biological source material [124]. In archaeology, sedimentary analyses feature centrally in the establishment of relative and absolute chronologies [125], with optically stimulated luminescence (OSL) dating being especially relevant in Pleistocene contexts [126]. Among other techniques traditionally used in archaeology, the microbial description of soils from ancient (ca. 550 BC) homes in western Sicily suggests that ancient human activities can be mirrored by specific activity-related soil microbial communities [127]. Evidence of past human behavior can be found in microbiological residues in soil microbiota (e.g., bacterial and fungal aDNA) as a record of anthropogenic modification.

In archaeological contexts, sedimentary ancient DNA (sedaDNA) occurs through the deposition of hair and skin flakes, bodily fluids (i.e., urine, blood, etc.), and also via eggshells, insect exuviae, regurgitation pellets, feathers, leaves, pollen, seeds, and living prokaryotes (through the secretion of plasmid and chromosomal DNA) and viruses present within organic matter [118]. Sedimentary and environmental samples have been a source for ancient viral [128], parasitic [129], and bacterial DNA [130,131].

Ancient DNA has changed our view of human evolution, from our relationship with other hominin groups (Neanderthals) and the existence of hominins that are difficult to analyze phylogenetically because of limited or no fossils (Denisovans and yet unknown hominins in Africa, respectively), to when and how different continents (Australasia, North and South America) were populated. It is reasonable to suggest that ancient pathogen DNA will have a similar impact on our view of the evolution of infectious diseases, with important implications for the prevention of emerging pathogens. Exploring the boundaries of host-pathogen divergence, including the maximum of pathogen divergence times in humans and nonhuman primates, and the ancient viral-host parallel evolutionary histories will help us to reconstruct the origin of present-day geographical distribution and diversity of clinical pathogen infections, and will hopefully allow us to foresee possible environmentally induced pathogen evolutionary responses.

Finally, while Africa continues to play a crucial role in preserving our ancestors’ valuable but limited paleontological and pathogen remains with the deepest evolutionary timescale, ancient DNA sampling in Africa is dominated by well-funded foreign laboratories. In order to give African scholars a role in the discovery and analysis of aDNA, a code of conduct to provide mutual collaborative research support should be standard in ancient DNA research. Moreover, ancient pathogen DNA research should be combined with modern clinical pathogen data and be equitable and provide advantages for all researchers worldwide, e.g., through shared data. The Nagoya Protocol on access to genetic resources and the fair and equitable sharing of benefits from the utility of such resources, including traditional knowledge, is a promising international platform and guideline for ethical and transparent benefit-sharing [132]. Moreover, it is encouraging to observe that research societies such as the Society of Africanist Archaeologists [133] see these issues as important and consider ethics, history, and the need for best practice in ancient DNA sampling for fruitful research and collaboration [134].
